# Spatial aspects of prebiotic replicator coexistence and community stability in a surface-bound RNA world model

**DOI:** 10.1186/1471-2148-13-204

**Published:** 2013-09-22

**Authors:** Balázs Könnyű, Tamás Czárán

**Affiliations:** 1 Department of Plant Systemtics, Ecology and Theoretical Biology, Eötvös Lorand University, H-1117 Pázmány Péter sétány 1/c, Budapest, Hungary; 2 Parmenides Foundation, Kirchplatz 1, D-82049 Munich/Pullach, Germany; 3 Hungarian Academy of Sciences, Eötvös Loránd University Research Group in Theoretical Biology and Evolutionary Ecology, H-1117 Pázmány Péter sétány 1/c, Budapest, Hungary

**Keywords:** Prebiotic replicators, Coexistence, RNA world, Parasite, Metabolic model, Prebiotic genome size

## Abstract

**Background:**

The coexistence of macromolecular replicators and thus the stability of presumed prebiotic replicator communities have been shown to critically depend on spatially constrained catalytic cooperation among RNA-like modular replicators. The necessary spatial constraints might have been supplied by mineral surfaces initially, preceding the more effective compartmentalization in membrane vesicles which must have been a later development of chemical evolution.

**Results:**

Using our surface-bound RNA world model – the Metabolic Replicator Model (MRM) platform – we show that the mobilities on the mineral substrate surface of both the macromolecular replicators and the small molecules of metabolites they produce catalytically are the key factors determining the stable persistence of an evolvable metabolic replicator community.

**Conclusion:**

The effects of replicator mobility and metabolite diffusion on different aspects of replicator coexistence in MRM are determined, including the maximum attainable size of the metabolic replicator system and its resistance to the invasion of parasitic replicators. We suggest a chemically plausible hypothetical scenario for the evolution of the first protocell starting from the surface-bound MRM system.

## Background

The principle of competitive exclusion is one of the robust ideas of theoretical ecology, stating roughly that biological entities whose existence and reproduction depends on the same common resource of limited supply will compete for that particular resource, and the kind of entity most effective in transforming resource to offspring will eventually displace all competing variants. The principle has been stated mathematically in many different forms [1,2], and can be applied to entities at very different levels of biological organization, from populations of individuals to populations of molecules. Infra-individual applications include competitive interactions of cancerous and normal cells within tissues, and also those of different chemical pathways “feeding” on the same chemical species, for example.

Since the principle applies to any self-reproducing entity using external resources of finite supply for multiplying itself, it is obvious that competitive exclusion could not be avoided even at times of the wake of life: the first self-replicating molecules (*prebiotic replicators*, of whatever chemical nature they were) inevitably competed for their own resources. They must have been modular too, in the sense of being assembled from an indefinite number of building blocks, each block belonging to one of a small set of chemically different species – *monomers*[3,4]. Since the number of modules in a replicator molecule is indefinite (at least in principle it is), the number of possible different sequences of monomers is infinite, warranting unlimited heredity [5] for prebiotic – just as for recent – macromolecular replicators.

To our present knowledge, the most likely candidate for the role of the early replicator – and thus for the molecular entity that took the first step towards life on Earth – is the RNA molecule consisting of four different monomers (*ribonucleotides*, [4,6,7]). The limiting common resource for these replicators was the supply of monomers which needed to be provided from external sources, at least at the early stages of prebiotic evolution. Therefore competitive exclusion was definitely a constraint on the diversity of any such “heterotrophic” RNA community: without other mechanisms maintaining the coexistence of different RNA species the molecular community would have been reduced to a single species by competition.

In the probable absence of specific protein catalysts at the wake of life, different sequences of prebiotic RNA molecules might have had diverse molecular functionalities like ribozyme activities [8-10] or transmembrane channelling of small molecules and ions [11], all of which were necessary for protocells (i.e., membrane-contained, metabolically active replicator assemblies capable of autonomous reproduction) to survive and reproduce. RNA diversity reduction due to competitive exclusion would have meant a massive loss of such functions. The maintenance of RNA diversity under prebiotic conditions is, therefore, one of the key issues of research on the origin of life.

There is a range of different suggestions in the theoretical literature of prebiotic evolution for solving the diversity problem [12-14]. One of them is based on the population dynamics (kinetics) of template-replicated molecules with complementary strands reversibly sticking together through nucleotide base-pairing. It can be shown [12,15-18] that such a system follows parabolic kinetics instead of the exponential that results from immediate and irreversible strand separation. Parabolic dynamics amounts to “the survival of everybody”, meaning that all competing sequences coexist even on a single common resource, independent of the function they might or might not have in a prebiotic entity – i.e., Darwinian selection and thus effective evolution is hampered in the parabolic system [16].

Other suggestions are built on the assumption of more or less obligatory cooperation among the RNA sequences feeding on the same limiting resource, i.e., on selective forces maintaining coexistence of different sequences in spite of the inevitable competitive interactions among them. The need for cooperation prevents the exclusion of any one replicator from such an RNA “community”, because it would severely decrease the fitness of the prebiotic “organism” to the functions of which it contributes [13,14]. The best known of these approaches is Eigen’s *hypercycle* model [19], in which replicators (proteins in this case) supply specific help to one another in a circular topology, so that each replicator receives direct catalytic aid for its replication from the previous member of the hypercycle, and gives a similar aid to the next one. The hypercycle is capable of maintaining the information content of all of its members as long as no mutations are allowed in the replicators. Mutations may produce two different types of parasitic sequences, however, which may destroy replicator coexistence: *shortcut* and *selfish* parasites [20]. The former directs its catalytic help to a member further away in the hypercycle instead of its immediate neighbour, thus reducing the length of the cycle by cutting it short. Repeated shortcut mutations will eventually reduce the system to a single replicator, thereby losing almost all the information content of the original system – that is, the result is the same as expected from competition without the hypercyclic organization of the replicators. The other type of harmful mutants (selfish parasites) would accept heterocatalytic help from one of the members of the hypercycle, but do not help effectively any other one. This kind of mutation cuts through the hypercycle, in effect changing it to a linear series of catalytic help with the selfish sequence as the terminal beneficiary, which results in the victory of the parasite and the fatal loss of information thereof. The spatially explicit (*cellular automaton*) implementation of the hypercycle model would be resistant to such selfish parasites, but not to shortcuts [21]. Its behaviour becomes complicated if the catalytic aid that replicators supply to each other are different [22].

**Figure 1:**
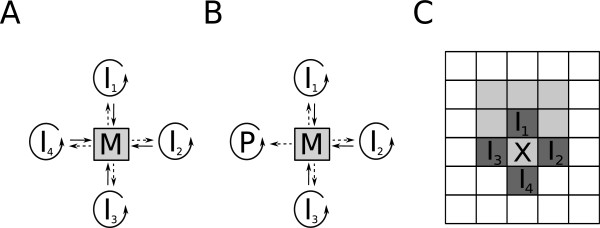
**The metabolic replicator concept and the neighbourhoods of the MRM model.** Panel **A:** The metabolic replicator system with four autocatalytic metabolic replicators (*I*_*i*_, *i* = 1, .., 4 within the circular arrows). *M* is the metabolic reaction network supported by the metabolic replicators as enzymes (solid lines) and producing monomers for their replication (dashed lines). Panel **B:** The relation of metabolic (*I*_*i*_, where *i* = 1..3) and parasitic (*P*) replicators to metabolism. Parasites consume monomers produced by the metabolic network but do not contribute to metabolism by catalytic activity. Panel **C:** Neighbourhoods defined in the MRM model. *X* is an empty site of the CA lattice, *I*_*i*_ (*i* = 1, .., 4) are the metabolic replicators. Dark grey sites are the replication neighbourhood of the empty site (*von Neuamnn neighbourhood* in this case) and light grey sites constitute the metabolic neighbourhood of replicator *I*_*1*_ (3×3 *Moore neighbourhood*).

Besides hypercyclic coupling there is another possible mechanism suggested for the obligatory cooperation of replicators which is capable of keeping the replicator community coexistent and evolvable. It is based on the idea that the catalytic help replicators give to each other needs not be provided in an “addressed” manner like it is within the ring of pairwise replication benefits in the hypercycle. Replicators can also cooperate indirectly, by collectively producing anything that serves the “common good” of the whole replicator community. The most straightforward way for them to do so is to contribute to the production of their own monomers, i.e., by becoming the enzymes of their own “metabolism” (Figure 1A). Both the Stochastic Corrector Model (SCM – [23]) and the Metabolic Replicator Model (MRM – [24]) are built on this assumption. Each replicator is the specific enzyme of a single reaction within a simple reaction network (metabolism) which produces monomers, the essential resource of replication for all the members of the community. Of course different “species” of replicators will compete for this single resource, but the competitive exclusion of any one of the metabolic enzymes is fatal for the whole community, causing one of the essential reactions of metabolism break down and thus stopping monomer supply. This is what actually happens in the mean-field approximation of the metabolic system [24]. The common principle of avoiding competitive exclusion in SCM and MRM models is imposing group selection on the cooperative replicator set, but the two approaches differ in how group selection is implemented. SCM assumes that the replicators are encapsulated in dividing membrane vesicles, the fitness of which depend on the sets of replicators they inherited from their parent vesicle through random replicator assortment. The daughter vesicles are selected for their metabolic efficiency: those containing complete sets of metabolic enzymes survive, others vanish. MRM does not assume membrane vesicles in the first place, but the replicators are thought to be bound to mineral surfaces which restrict their movement and thus represent a viscous substrate that keeps parent and offspring close to each other. This has been shown to maintain the coexistence of the replicator community [24] and also to provide effective defence against parasitic mutants which, both in SCM and in MRM, are replicators not contributing to metabolism (Figure 1B) but feeding on the monomers it supplies [23-26]. MRM offers an additional advantage: the inevitable but not fatally deleterious presence of parasitic replicators opens the way for the straightforward mutational conversion of parasitic replicators into new metabolic enzymes or catalysts providing other chemical functions beneficial for the cooperating replicator set [27]. Such benefits may include the production of membrane units, which may lead to the intrinsic emergence of vesicular compartmentalization of the replicator system, and thus to the evolution of free-living protocells which are not tied to the mineral surface any more. We have also shown that the adaptive increase of efficiency and specificity of enzymatic replicators (e.g., ribozymes) occur naturally within the MRM framework [28].

This paper is a detailed study of the spatial aspects of replicator evolution within the MRM framework. We used an updated implementation of the MRM model also described in [27], in order to perform a thorough simulation investigation of the parameter space of the model – a task that could not be performed earlier due to the lack of appropriate computer capacity at the time. The spatial parameters of the model are intimately connected to our implicit assumptions on the physical-chemical criteria of replicator coexistence, because they are proxies to habitat viscosity and to the spatial ranges of competitive and mutualistic interactions between replicator molecules, which have been shown to be key factors in the maintenance of metabolic cooperation in MRM [24]. The specific questions we wish to answer with the model are related to the effects of replicator (macromolecular) mobility and metabolite/monomer diffusibility on system persistence (i.e., replicator coexistence) and on the attainable maximal size of the system which could not be systematically investigated in previous studies. How these space-related effects are implemented in the model is explained in the next section.

## Methods

The general assumptions of all MRM systems [24,27,28] are the following:

1) *The chemical identity of early replicators*. The MRM framework does not make any explicit assumptions with regard to the chemical identity of prebiotic replicators, but straightforward general principles constrain the possibilities to modular (and, consequently, digital) structures capable of unlimited heredity [5]. These constraints practically exclude the majority of known chemical entities from among the plausible molecule types, except for variants of recent nucleic acids and proteins [4,29]. According to speculations Kauffman’s hypothesis of “collectively autocatalytic sets” [30,31] random sets of oligopeptides connected by pairwise heterocatalytic interactions are possible candidates for a proper evolvable system of digital replicators, but most students of the origin of life today agree that RNA, or RNA-like molecules are by far the most likely entities responsible for booting up life on Earth 3–4 billion years ago [4,6,7,9]. The MRM system is built on the RNA world scenario allowing for some chemical variations but maintaining the postulates of a modular, template-replicated macromolecule as the basic chemical entity of prebiotic evolution. As an initial simplifying assumption we assume that the sister strands of replicating RNA molecules are identical; the complications arising from complementary strands (i.e., that of the genotype-phenotype distinction in template replication) will be studied in detail in another paper.

2) *The role of mineral surfaces*. Experimental data of very different nature provide strong indirect evidence for the assumption that mineral underwater surfaces (rocks of pyrite, clay minerals like montmorillonite, etc.) may have played a decisive role in the evolution of prebiotic replicators. Such mineral surfaces may have acted as catalysts for nucleotide binding [32]; they might be responsible for the homochirality of biomolecules [33,34]; they might have assisted membrane production and thus the formation of the first protocell vesicles [35]; and they may have protected replicators from the harmful effects of UV radiation [36]. Therefore we have adopted the assumption that the most probable arena for prebiotic evolution may have been on such mineral surfaces which can bind RNA molecules reversibly through divalent cations [37]. Detachment and re-attachment of parts of the macromolecules results in their caterpillar-like movement on the surface, which is in turn responsible for their limited rate of spatial mixing – a feature that will be shown to be of crucial importance later.

3) *Enzymatic activity of replicators*. Many recent RNA molecules take part in several vital chemical processes of recent cells as catalysts (*ribozymes,*[10]). Early prebiotic RNA world systems must have relied mostly on the catalytic potential of ribozymes, because translation and thus more efficient protein enzymes are later achievements of evolution. The broad catalytic potential of RNA molecules was justified in different independent experimental studies [8,9,38,39]. Moreover, Biondi and his co-workers could experimentally demonstrate that surface-bound replicators do not lose their enzymatic activities [36].

4) *Metabolism*. The main assumption of the MRM is that each member of a set of different replicator types (i.e., replicators of different nucleotide sequence) catalyses a single reaction in a hypothetical metabolic reaction network in which their own building blocks (monomers) are produced. Therefore monomers for replication are self-supplied only in the presence of a complete set of metabolic replicators (Figure 1A). We assume that metabolism works on a local scale, so that all replicators required for metabolism to produce monomers must be in close spatial proximity to each other, otherwise metabolism breaks down locally. The spatial range within which all metabolic ribozymes need to be present is called the *metabolic neighbourhood* (Figure 1C). A replicator in the centre of a metabolically complete neighbourhood has a chance to replicate – others do not. Notice that we do not yet assume any explicit topology and/or stoichiometry for the metabolic reaction network here, even though it might be of substantial effect on the actual dynamics of the metabolic replicator system. We are studying this aspect of the dynamics of MRM in another modelling project.

5) *Metabolites*. The chemical nature of precursors, intermediary metabolites and monomers is completely disregarded in the MRM system, just like the topology of the metabolic reaction network itself. What we implicitly consider are a few general features of small molecules in relation to their movement on and detachment from the mineral surface. We assume that small molecules move on the surface faster than macromolecules do, and they can desorb from the surface with a probability higher than replicators. Both of these assumptions reflect that small molecules are certainly less attached to the surface than the macromolecules built from them (or from similar small molecules). Metabolite diffusion and desorption are implemented through the size of the metabolic neighbourhood (Figure 1C), the radius of which is proportional to the average distance that a small molecule can cover before it desorbs from the surface or is consumed in a replication process.

6) *Error-free replication*. The most difficult “missing link” and at the same time the least experimentally accessible aspect of the MRM approach is that of RNA replication. The sequence of a relatively simple RNA-dependent RNA polymerase ribozyme has not been discovered so far, but of course any RNA World model should be able to account for the replication of RNA molecules in order to explain the evolution of RNA molecules within the RNA World scenario. Evolving such a replicase ribozyme is one of the biggest challenges for recent *in vitro* RNA evolution experiments [40-42]. Lacking an efficient RNA replicase ribozyme we need to assume for the time being that the template replication of RNA molecules was nevertheless possible at the time of the wake of life, either because there was a – so far undiscovered – replicase ribozyme present in the RNA world after all, or because RNA replication was – however weakly – catalysed by some unknown agent or the mineral surface itself ([32]; see point *1*). A minor difficulty arises from the omission of the fact that any template replication is prone to mismatch errors (mutations) resulting in copies slightly different from the template. In fact this is the *error catastrophe* problem that the coexistence models of prebiotic evolution (i.e., the hypercycle – [19] –, parabolic growth models – [12] – and MRM – [24] – systems) are meant to solve in the first place, but it is essentially solved by the assumption that the genetic information to be transmitted is split into short sequences. Therefore MRM makes the simplifying assumption that RNA replication is error-free on the ecological time scale for which the coexistence of metabolic replicators is investigated. Alternatively, we may assume that the replication rates of the different replicator types are renormalized to account for the mutational loss into non-functional RNA forms.

7) *Double-strand separation and local dispersion*. Another difficulty related to the problem of experimental RNA replication is that even if the complementary strand can be formed, the copy cannot be separated from the template without imposing chemical conditions on the system that are very far from any reasonable assumption of prebiotic environmental conditions [5,18]. For lack of empirical knowledge on this issue we are again forced to assume that strand separation does occur somehow due to a mechanism so far unknown. The sister strands are assumed to be identical and to remain spatially close to each other, i.e., replicator dispersal is local. The problem of complementary strands (i.e., that of the genotype-phenotype distinction in template replication) will be studied in detail in another paper.

### Model details

#### The model framework

The MRM system is implemented as a stochastic cellular automaton (SCA). The mineral surface on which the reactions (metabolism and replications) take place is represented by a square lattice of sites, with each site is capable of binding one replicator molecule at most. The opposite margins of the lattice are merged forming a toroidal structure to avoid edge effects. Assuming *n* different replicator types the number of possible different states for a site is *n* + 1, including the “empty” state and the states “occupied by replicator type *i* (*i* = 1, … , *n*)”. The model is initiated with a random community of *n* = 4 different replicator types occupying 80% of the sites at *t* = 0. The updating algorithm is random: the state of each site is updated once per time unit on average, in a random order *(asynchronous updating rule)*. One generation (*t* to *t* + 1) consists of such elementary random update steps equal in number with the number of sites in the lattice. The lattice size we used throughout the simulations was 300 *×* 300, i.e., one generation consisted of 90.000 updates.

#### Replication and decay

The next state (at time *t* + 1) of a site depends on the actual state (at time *t*) of itself and of its neighbours. Empty and occupied sites are updated by separate algorithms: “empty” sites can become occupied with probability *p*_*f*_ by a copy of replicator *f* from within the *replication neighbourhood* (Figure 1C) of the empty site, whereas occupied sites can turn into the “empty” state (replicator decay) with the decay probability *p*_*d*_. Decay probability *p*_*d*_ is a constant, but the replication probability *p*_*f*_ of the replicator molecule *f* depends on the actual replicator composition of its own *metabolic neighbourhood* (Figure 1C) and that of its competitors (i.e., replicators from the *replication neighbourhood* of the same empty site). The individual “claim” *C*_*f*_ of the replicator *f* for occupying the empty site depends on its monomer supply *M*_*f*_ and its type-specific replication rate *k*_*f*_ as

(1) $$C_{f}=k_{f}\;M_{f},$$

and

(2) $$M_{f}=\sqrt[{n}]{\prod_{i=1}^{n}x_{i}\left(f\right).}$$

*x*_*i*_(*f*) is the number of type *i* replicators within the metabolic neighbourhood of focal replicator *f*, and *i* runs through all replicator types needed to catalyse the metabolic reactions. Thus, the local monomer supply of the focal replicator *f* depends on the presence of *all* metabolic replicators within its own metabolic neighbourhood – any one of the *n* metabolic replicator types missing means that the corresponding *x*_*i*_(*f*) = 0 and thus also *M*_*f*_ = 0. This in turn implies no local monomer production and therefore no chance of replication for the focal molecule *f*. Each replicator within the replication neighbourhood of an empty site has a chance

(3) $$p_{f}=\frac{C_{f}}{C_{e}+\sum_{m}C_{m}}$$

to occupy the empty site with a copy of itself; *m* runs through all replicators within the replication neighbourhood of the focal replicator *f*, and *C*_*e*_ is a constant representing the claim of the empty site for remaining empty. Obviously, the probability that the empty site remains empty is

(4) $$p_{e}=\frac{C_{e}}{C_{e}+\sum_{m}C_{m}}$$

#### Replicator diffusion

The movement of replicators on the mineral surface is implemented with the Toffoli-Margolus algorithm: randomly chosen 2×2 blocks of sites are rotated 90° left or right with equal (0.5) probability [43]. The intensity of replicator diffusion is scaled by the average number *D* of diffusion steps per site per generation. Note that even *D* = 0 means some minimum mixing of replicators on the surface, due to the fact that each newborn copy is placed into a site different from – adjacent to – the one occupied by the template [28].

#### Parasites

The only parasitic replicator of the MRM system is the one that consumes the monomers produced by cooperating metabolic replicators but does not contribute to monomer production itself at all (Figure 1B). Since the secondary structure – which is responsible for replication speed – of such parasites is not constrained by any functional criteria like metabolic efficiency we assume that the replication rate *k*_*p*_ of parasites is the highest of all replicator types in the system.

## Results

We have performed a systematic simulation study to reveal the effects of changing the model parameters critical for the coexistence of the replicators. Since the mean-field approximation (i.e., the well-mixed version) of the MRM system is not coexistent (c.f. [24]) the spatial aspects of the present model are of crucial interest from the viewpoint of system persistence and stability. We focused our interest on three parameters which are separated into two groups: those related to 1) the mobility of replicators (the size of the replication neighbourhood (*r),* and mobility of replicators on the surface, *D*); and 2) to metabolite/monomer diffusibility on the surface (the size of metabolic neighbourhoods, *h*). Other parameters were kept constant throughout the simulations. Lattice size was *L* = 300×300; simulations were initiated with *n* = 4 replicator types randomly assigned to 80% of the sites at *t* = 0. The decay rate was *d* = 0.2, the claim of empty sites for remaining empty was *C*_*e*_ = 2.0, and the replication rates of the four different replicator types were *k*_*1*_ = 3.0, *k*_*2*_ = 5.0, *k*_*3*_ = 7.0 and *k*_*4*_ = 9.0. In simulations with parasitic replicators present the parasite was the fourth type added to the community of three metabolically cooperating replicators; the replication rate of the parasite was *k*_*p*_ = 9.0.

The model was coded in *C*, compiled with *gcc* (*GNU C Compiler* 4.4.5) and run under Linux (Debian 6.0.1). For each parameter set we have produced 5 replicate runs with different random number sequences. The conclusions of a long series of batch simulations are the following:

### The effects of local monomer production/consumption and limited replicator diffusion

**Figure 2:**
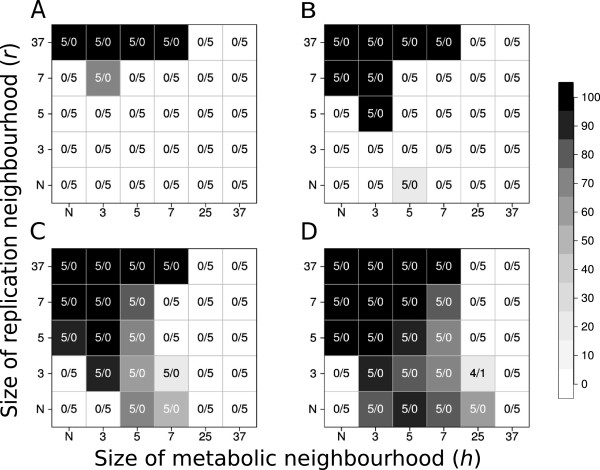
**Coexistence of metabolic replicators as the function of replicator diffusion (*D*), metabolic (*h*) and replication (*r*) neighbourhood size.** The panels of the figure differ in the number of diffusion steps per generation: Panel **A: ***D* = 0, Panel **B: ***D* = 1, Panel **C: ***D* = 4 and Panel **D: ***D* = 100. *x*- and *y*-axes are the sizes of metabolic neighbourhoods (*h*) and replication neighbourhoods (*r*) respectively (*N*: *von Neumann* neighbourhood; 3: *3×3*, 5: *5×5*, 7: *7×7*, 25: *25×25* and 37: *37×37 Moore* neighbourhoods). The grayscale shades correspond to average replicator densities (%) on the whole grid at the end of the simulations (i.e., for *t* = 1.000). The numbers within panels indicate coexistent/extinct replicate simulations out of the five repetitions with the same parameter set and different pseudo-random number sequences.

Figure 2 shows simulation results of the MRM at different sizes of the replication neighbourhood (*r*) and the metabolic neighbourhood (*h*), with different values of the replicator diffusion parameter (*D*) at a fixed system size (*n* = 4). The main effects showing up on Figure 2 are:

a) system persistence and total replicator population densities depend on all three space-related model parameters (*r*, *h* and *D*);

b) increasing replication neighbourhood size (*r*) or replicator diffusion (*D*) or both are advantageous for persistence and population density;

c) persistence and population density follow optimum courses with the size of the metabolic neighbourhood (*h*): too small and too large *h* are both fatal for the system;

d) persistent systems attain high population densities;

e) the results are robust with respect to persistence: 5 replicate runs almost always (with only a single exception) produce the same outcome (with low standard deviations): either always persistence or always extinction, depending on the actual parameter set. Note that the replicator populations reach their equilibrium densities during the simulations of 1.000 generations each.

### The effect of spatial parameters on the maximum attainable system size

**Figure 3:**
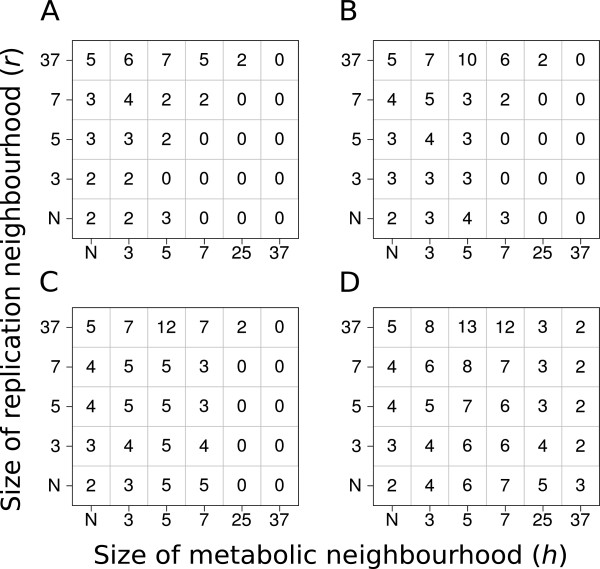
**The maximum number of coexisting metabolic replicators as the function of replicator diffusion (*D*), metabolic (*h*) and replication (*r*) neighbourhood size.** The panels of the figure differ in the number of diffusion steps per generation: Panel **A: ***D* = 0 , Panel **B: ***D* = 1, Panel **C: ***D* = 4 and Panel **D: ***D* = 100 *x*- and *y*-axes are the sizes of metabolic neighbourhoods (*h*) and replication neighbourhoods (*r*) respectively (*N*: *von Neumann* neighbourhood; 3: *3×3*, 5: *5×5*, 7: *7×7*, 25: *25×25* and 37: *37×37 Moore* neighbourhoods). The numbers within panels show the maximum number of coexisting metabolic replicator types (*n*_*max*_ *= q*) for the given parameter set. Other parameters: *p*_*d*_ = 0.2, *C*_*e*_ = 2.0, *k*_*i*_ = 3.0 + 2.0*i* (*i* = 0, .., *max*). (*max*. is the maximal number of replicators that can be seen within a square on the panel).

We have tested the MRM system for the maximum number of metabolic replicator types (i.e., the largest system size *n*_*max*_ *= q*) that can coexist at different parameter sets. The results are condensed into Figure 3, with the following conclusions:

f) *q* follows a course with increasing *r*, *h* and *D* similar to that of system persistence and total population density at *n* = 4: increasing *r* and *D* are beneficial, too low and too high *h* are adverse for the maximum number of coexistent replicators;

g) within the parameter range tested the largest system size can go up to about *n*_*max*_ = 13 different replicator types under optimal conditions.

### The effect of parasites

**Figure 4:**
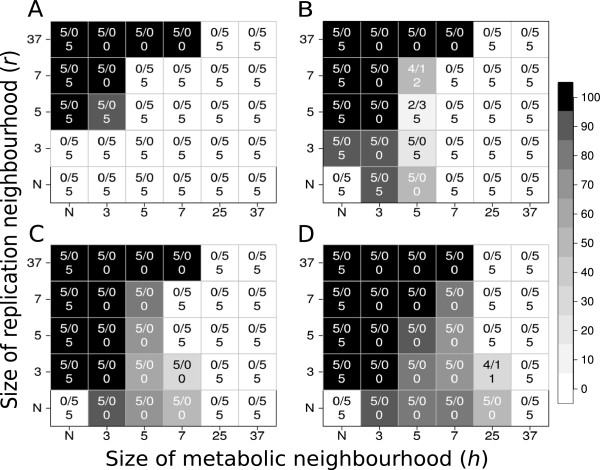
**Coexistence of metabolic replicators and a parasitic one as the function of replicator diffusion (*D*), metabolic (*h*) and replication (*r *) neighbourhood size.** The panels of the figure differ in the number of diffusion steps per generation: Panel **A: ***D* = 0, Panel **B: ***D* = 1, Panel **C: ***D* = 4 and Panel **D: ***D* = 100. *x*- and *y*-axes are the sizes of metabolic neighbourhoods (*h*) and replication neighbourhoods (*r*) respectively (*N*: *von Neumann* neighbourhood; 3: *3×3*, 5: *5×5*, 7: *7×7*, 25: *25×25* and 37: *37×37 Moore* neighbourhoods). The grayscale shades correspond to average replicator densities (%) on the whole grid at the end of the simulations (i.e., for *t* = 1.000). The numbers within panels indicate coexistent/extinct replicate simulations out of the five repetitions with the same parameter set and different pseudo-random number sequences. The third number is the number of replicate simulations in which the parasite died out.

Figure 4 shows the results of a series of simulations with all the parameters set to exactly the same values as in the simulations that produced Figure 2, except that the 4th replicator is a parasitic one: it does not contribute to metabolism at all, but uses the product of metabolism – i.e., monomers – for its own replication (Figure 1B). The parasite is the fastest of the four types in replication, with *k*_*p*_ = 9.0 (compared to *k*_*1*_ = 3.0, *k*_*2*_ = 5.0 and *k*_*3*_ = 7.0 of the cooperating types). Figure 4 suggests the following conclusions:

h) replacing a metabolic cooperator with a parasite does not do much harm to the metabolic system as a whole: the parameter range of coexistence does not shrink. (In fact it expands in this case, but this is due to the simultaneous decrease of system size from *n* = 4 to *n* = 3 – see the Discussion for an explanation);

i) at very small metabolic neighbourhood sizes the parasite may be expunged from the metabolic system completely;

j) increasing the mobility of the replicators (i.e., larger values of *D* and/or *r*) favours the parasite in terms of its chances of survival and equilibrium abundance, but even at high replicator mobility the parasite is unable to exclude metabolic cooperators and to ruin the metabolic system;

k) the parasitized metabolic system is also robust with respect to persistence: only a few borderline cases deviate from unequivocal coexistence or unequivocal extinction in 5 replicate simulations.

## Discussion

The most obvious (and also the most important) feature of the spatially explicit system of metabolic replicators is that it maintains stable replicator coexistence within a large part of the physico-chemically feasible section of its parameter space, and it does so in a surprisingly robust manner (cf. Result e) above), especially compared to its mean-field (non-spatial) approximation which is never coexistent [24]. We start the discussion of the model outputs by explaining the mechanisms that maintains coexistence in terms of the assumptions of the model; then we discuss why we think that these assumptions apply at the physico-chemically feasible part of the parameter space, and conclude with explaining why metabolic parasites represent only a moderate threat and a potentially beneficial pre-adaptation for the system.

### Replicator coexistence through the local advantage of rarity

The long-term coexistence of different replicators which are capable of exponential population increase is possible only through regulated population dynamics of the replicator species present [2]. Regulation must act through the negative feedback of population abundance on population growth. This means that high abundance must be a disadvantage for the growth rate (*fitness*) of the common type and low abundance must be advantageous, so that rare species of replicators must enjoy a relative edge in terms of their growth rate compared to common ones. Only this *advantage-of-the-rare* mechanism can ensure that all different types of replicators remain coexistent despite their different replication rates (*k*_*i*_). In the metabolic replicator system the advantage of rarity is realized through the compulsory metabolic cooperation of the replicator community, since the replicators collectively produce their own resources for replication – see Assumption 4), Figure 1A and Equation 2 above. All metabolically active replicator types must be present with at least one copy within a distance that the metabolites (precursors, intermediary compounds and monomers) can easily cover by surface diffusion. This distance is represented by *h*, the radius of the metabolic neighbourhood. Copies of a rare replicator type enjoy the advantage of having a higher chance to be complemented by the more common species within their metabolic neighbourhood than the other way round. This effect depends on *h*, the size of the metabolic neighbourhood following an optimum course (Result c). Very small metabolic neighbourhoods may not be large enough to accommodate a sufficient number of replicators: e.g., the von Neumann neighbourhood consists of 5 sites which, of course, cannot contain more than 5 different replicator molecules (Figure 3). This limits system size to *n*_*max*_ = 5, but the chance of metabolic complementation may be very small in the von Neumann neighbourhood even for *n* = 4 or 3, especially if replicator mobility (i.e., replicator diffusibility *D* and/or *r*, the size of the replication neighbourhood) is small (cf. Result b, Figure 3). On the other hand, large metabolic neighbourhoods decrease the advantage of rarity, because the chance of metabolic complementation increases with *h* faster for common replicator species than for rare ones. In fact increasing the size of the metabolic neighbourhood means approaching the mean-field case (with respect to metabolic interactions) in the limit: we arrive at the mean-field interaction scheme with *h* = *L* (i.e., at lattice size). We have found that moderately small metabolic neighbourhoods are optimal for coexistence (Result c), for the overall equilibrium density of replicators (Result d) and for the maximum of system size *q* (Results f and g) alike. Attainable system size, i.e., the genetic information content of an evolving prebiotic replicator system is a crucial problem of research on the origin of life [19,44-47]. Our model predicts that the maximum number of metabolic replicators would be dependent on the spatial mobilities (diffusion) of the surface-bound replicators and the metabolites (Figure 3), and that physically feasible values of mobility can maintain a substantial number of different replicators – and thus a sufficient amount of genetic information – in the Metabolic Replicator Model.

**Figure 5:**
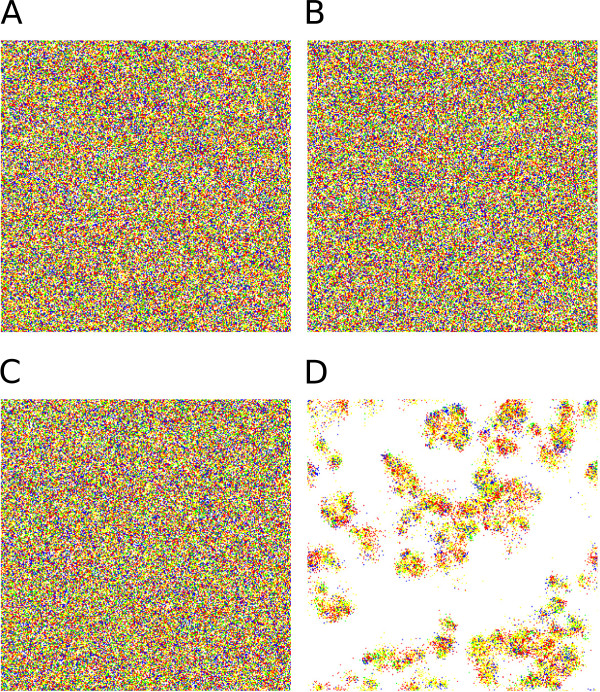
**Snapshots of the CA lattice pattern of the replicator population on the surface.** Panels **A-B:** The typical homogeneous pattern of replicators at the beginning (Panel **A**, *t* = 0) and at the end (Panel **B**, *t* = 1.000) of the simulations. Parameters: *D* = 100, *h* = von Neumann neighbourhood and *r* = 5x5 Moore neighbourhood. Panels **C-D:** The pattern of replicators at the beginning (Panel **C**, *t* = 0) and at the end (Panel **D**, *t* = 1.000) of another simulation with different parameters allowing for a sparse metabolic system: *D* = 1, *h* = 5x5 Moore neighbourhood and *r* = von Neumann neighbourhood. Colour code: white – empty sites, green – metabolic replicator 1 with *k*_*1*_ = 3.0, blue – metabolic replicator 2 with *k*_*2*_ = 5.0; red – metabolic replicator 3 with *k*_*3*_ = 7.0 and yellow – metabolic replicator 4 with *k*_*4*_ = 9., *k*_*i*_*-s* are the replication rates of metabolic replicators.

Since the presence of all metabolically active replicators within relatively small neighbourhoods is necessary for metabolism to work, a substantial measure of spatial mixing of the replicators is necessary for system persistence. Increased mixing can be achieved in two different ways: either the sister strands need to be placed relatively far apart in the lattice after replication (i.e., the size of the replication neighbourhood, *r*, should be relatively large) or the diffusive movement of the replicators on the mineral surface need to be faster (i.e., *D* should be large). Indeed, increasing any one or both of these parameters enhances coexistence and increases overall replicator density (Result b). As a consequence, the spatio-temporal pattern of a viable replicator community always lacks conspicuous mesoscopic structures like spiral waves [21]; the visual impression of a persistent, dense metabolic system is a homogeneous mix of all the different metabolic replicator types (Figure 5A and B). Patchy structures may develop in sparse persistent systems (Figure 2B), but the patches themselves are still homogeneous mixes of all the metabolic replicator species even then, scattered within the matrix of empty regions (Figure 5C and D).

### The physical interpretation of metabolic neighbourhood size

**Figure 6:**
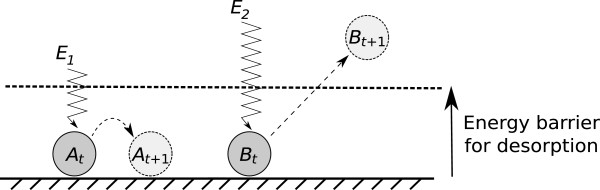
**Desorption of small molecules from the surface.** The surface binds molecules *A* and *B* at time *t*. Energy input might detach them from the surface and they either reattach at another location (*A*) or leave the surface (*B*) by time *t +* 1, depending on the energy input (*E*_*1*_ *< E*_*desorption threshold*_ < *E*_*2*_).

The key parameter in our phenomenological model on the metabolic cooperation of different replicator species is metabolic neighbourhood size *h*. Loosely speaking, *h* represents the maximum distance across which the replicators can pass metabolites to each other between the reactions they catalyse on them, and also the maximum distance from which the monomers – the end product of the metabolic reaction network – can be recruited by the template strand for its replication. Everything else kept constant, the distance that a single particle (like a monomer molecule or other small metabolites) can cover by surface diffusion depends on two factors: the strength of the adherence of the particles to the surface which determines the average time that particles spend on the surface before desorption, and the speed at which they can move horizontally (Figure 6). These two factors must be correlated: strong affinity to the surface implies slow diffusion and relatively long time spent on the surface before desorption, whereas weak affinity means faster diffusion and a short time before particle desorption. The distance covered by surface diffusion is limited in both cases. Assuming that desorbed particles are lost for metabolism (metabolically active ribozymes being tied to the surface), all this means that for metabolism to be able to produce monomers for replication a complete set of metabolically active replicators must be within average particle diffusion distance to each other. That distance is represented by *h* in the model.

Note that we have assumed relatively fast mixing for the replicators themselves on the same surface, which requires that the adherence of the replicators to the surface be moderate. Since any strength of surface adherence implies a limited distance of metabolite diffusion, this criterion does not contradict that of a small to moderate *h*. However, weak surface affinity means a high loss rate of metabolites from the surface, which in turn requires that the system be fed from a rich source of precursor molecules. *Black smokers* are often mentioned as possible candidates for supplying the necessary high concentration of precursors [48,49]. Of course, these assumptions call for verification both empirically and theoretically. As for the theoretical part, we are preparing a manuscript that takes the details of metabolite and replicator diffusion into account in a chemically more explicit spatial model (Kőrössy & Czárán, in prep.).

### Metabolic parasites – regulated coexistence with the metabolic system

The metabolic replicator community cannot escape being parasitized by mutant replicators which do not contribute to metabolism but use its product (the monomers) for their own replication. However, parasites of even very high replication rates do not ruin the cooperation of metabolic replicators but coexist with them indefinitely (Result h). The reason for this is the complement of the advantage-of-the-rare mechanism maintaining the coexistence of metabolically active replicators, namely the *risk-of-the-common*: Whichever replicator becomes too abundant has a high chance of finding itself in a metabolically incomplete metabolic neighbourhood and thus short of monomers for its replication. This is especially the case for parasites which do not even play a role in monomer production. Therefore, local replicator assemblies dominated by the parasite are doomed to local extinction, preempting the surface for the invasion of metabolically complete, expanding local communities. In fact the emergence of parasitic mutants is unavoidable, but it does not substantially change the chances of survival for the metabolic system as a whole, because the density of parasites is kept at low or at least at tolerable levels by the local regulatory mechanism just described. In the model we have replaced a metabolically active, cooperating replicator with a parasitic one, which resulted in an increased chance of coexistence, but this effect was due to the fact that the size of the system fell from *n* = 4 to *n* = 3 in the specific case modelled. Smaller system size is, of course, advantageous for system persistence, because it is easier to maintain complete metabolic neighbourhoods of small radii (i.e., of small *h*) with fewer replicator types required to cooperate (Result h). Note, however, that an established metabolic replicator system cannot afford the complete loss of an essential metabolic replicator without the collapse of the whole system: very strong selective forces would act against such changes.

The only way to eliminate the parasite completely from the metabolic system is to decrease metabolic neighbourhood size *h* to the extreme (Result i). The regulatory power of the spatial (local) risk-of-the-common mechanism is perhaps best illustrated by this result, since the parasite, which is of the *highest* replication rate *k* of all the replicators present, is the only one that disappears from the system at very small *h*. For the parasitic replicator to persist it needs to gain access to the monomers produced by the cooperators, which in turn requires that metabolic neighbourhood size *h* (i.e., the distance to which monomers can diffuse before desorption from the surface) and/or the replicator mobility parameters *D* and *r* be sufficiently large (Result j). These conclusions are also robust: repeated simulations consistently produce the same outcomes (Result k).

**Figure 7:**
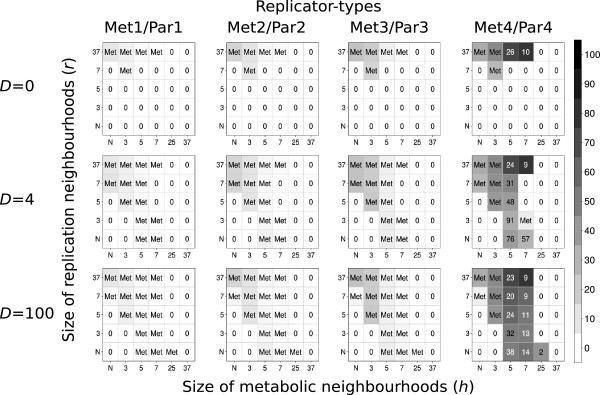
**Coexistence of metabolic replicators with several parasitic replicator species in MRM.** The rows of the figure differ in the number of diffusion steps per generation: first row of panels: *D* = 0, second row: *D* = 4; third row: *D* = 100. *x*- and *y*-axes are metabolic (*h*) and replication (*r*) neighbourhood sizes respectively (*N*: *von Neumann* neighbourhood; 3: *3×3*, 5: *5×5*, 7: *7×7*, 25: *25×25* and 37: *37×37 Moore* neighbourhoods). The grayscale shades of the cells correspond to average replicator densities (%) on the whole grid at the end of the simulations (at *t* = 1.000). The numbers within the cells of the panels indicate the average ratio of the number of metabolic replicators to the number of parasitic replicators at the end of the simulations (Met_x_/Par_x_, *x* = 1, .., 4) in five replicate runs of the simulation program with each parameter set. *Met* indicates that the parasitic replicator type died out; zeros mean that the system collapses (no replicator survives to *t* = 1.000). Replication rates: *k*_*1m*_ = 3.0, *k*_*1p*_ = 4.0, *k*_*2m*_ = 5.0, *k*_*2p*_ = 6.0, *k*_*3m*_ = 7.0, *k*_*3p*_ = 8.0, *k*_*4m*_ = 9.0 and *k*_*4p*_ = 10.0; subscripts *m* and *p* denote metabolic and parasitic replicator types, respectively.

Note that replicators of even very different monomer sequences are *functionally equivalent parasites* if they do not contribute to monomer production, therefore parasites constitute a structurally heterogeneous but functionally homogeneous, persistent population strictly regulated by the metabolic system itself. In conclusion, it seems reasonable to claim that for realistic monomer and replicator mobility parameters the metabolic replicator system will maintain a viable cooperator community with an efficiently downregulated, structurally diverse but functionally uniform parasite population. Out of several parasite species differing in their replication rates only the fastest one (of highest *k*_*p*_) survives, all other parasitic species go extinct (Figure 7).

### Metabolic parasites as preadaptations to protocell evolution

**Figure 8:**
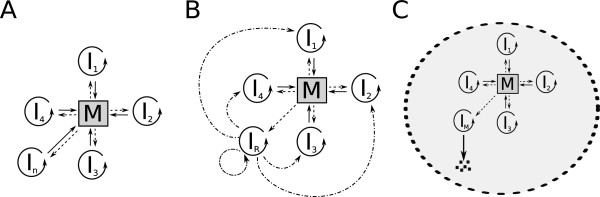
**Possible evolutionary improvements of the metabolic replicator model.** Panel **A:** Evolution of a new metabolic replicator (*I*_*n*_). Panel **B:** Evolution of the replicase replicator (*I*_*R*_*,* dashed-dotted lines represent the replicase activity). Panel **C:** Evolution of an enzymatic replicator producing protocell-membrane elements (*I*_*M*_, the thick dashed line represents the membrane, and squares are the membrane units). *I*_*i*_ (*i* = 1, .., 4) are the metabolic replicators on all panels and *M* stands for the metabolic reaction network. Solid, dashed and dotted-dashed arrows are the same as in Figure 1.

Since the parasites are always present in the metabolic replicator system and they are subject to nearly neutral mutations without strong selection constraints, the parasite population can roam through the sequence space, and occasionally “discover” sequences that may provide some, even if very small, advantage to the metabolic system as a whole. The advantage may be metabolic (the mutant may serve the system as a new enzymatic replicator or a co-enzyme of an existing one, Figure 8A), but it may be of any other nature like contribution to the replication of metabolic replicators (i.e., evolving replicase activity, Figure 8B, [27]) or the spatial separation of the metabolic system from its surroundings (i.e., evolving into enzymatic replicators producing protocell-membrane elements, Figure 8C). If the service offered by the beneficial mutant helps the system in any way to increase faster than before it occurred, then the mutant will spread over the whole surface. Such “domesticated” parasites are then further evolved by the system itself to be more efficient in their beneficial function, possibly until the new trait becomes an essential feature of the system. The theoretical exploration of this – in its very essence group-selectionist – scenario of prebiotic evolution requires a series of chemically more explicit simulation studies, part of which are underway.

## Conclusion

The Metabolic Replicator Model is one of the theoretical approaches attempting to set up a feasible scenario of the origin of life based on the RNA World paradigm. The MRM system is consistent with most of the known empirical facts regarding prebiotic systems chemistry, and it can explain the coexistence of different RNA-like replicator macromolecules. The central assumption of the model is that the replicators – besides carrying genetic information in their monomer sequences – have phenotypes as well: they act as the enzymes of a simple metabolism producing monomers for their own replication. The consequent indirect mutualism between the replicators allows for their coexistence and makes the system resistant to parasitic replicators. In this paper we have explored the parameter space of the MRM system, with the main emphasis on the maximum number of potentially coexistent replicators under different mobility regimes of the macromolecules and metabolites on a mineral surface. The stable MRM system is also capable of evolving more complex enzyme functions like that of a replicase or membrane synthesis – this leaves the possibility for MRM to evolve towards membrane-coated self-reproducing vesicles (*protocells*) open.

## Competing interests

The authors declare that they have no competing interests.

## Authors’ contributions

BK and TC designed, analysed and interpreted the study. Both authors contributed to writing the manuscript and approved the final version.
